# The Prognostic 97 Chemoresponse Gene Signature in Ovarian Cancer

**DOI:** 10.1038/s41598-017-08766-5

**Published:** 2017-08-29

**Authors:** Abel Matondo, Yong Hwa Jo, Muhammad Shahid, Tae Gyu Choi, Minh Nam Nguyen, Ngoc Ngo Yen Nguyen, Salima Akter, Insug Kang, Joohun Ha, Chi Hoon Maeng, Si-Young Kim, Ju-seog Lee, Jayoung Kim, Sung Soo Kim

**Affiliations:** 1 Department of Biomedical Science, Graduate School, Kyung Hee University, Seoul, Republic of Korea; 2 Department of Biochemistry and Molecular Biology, Medical Research Center for Bioreaction to Reactive Oxygen Species and Biomedical Science Institute, School of Medicine, Kyung Hee University, Seoul, Republic of Korea; 3 Department of Medical Oncology and Hematology, School of Medicine, Kyung Hee University, Seoul, Republic of Korea; 4 Department of Systems Biology, Division of Cancer Medicine, University of Texas MD Anderson Cancer Center, Houston, Texas USA; 5 Departments of Surgery and Biomedical Sciences, Cedars-Sinai Medical Center, Los Angeles, CA USA

## Abstract

Patient diagnosis and care would be significantly improved by understanding the mechanisms underlying platinum and taxane resistance in ovarian cancer. Here, we aim to establish a gene signature that can identify molecular pathways/transcription factors involved in ovarian cancer progression, poor clinical outcome, and chemotherapy resistance. To validate the robustness of the gene signature, a meta-analysis approach was applied to 1,020 patients from 7 datasets. A 97-gene signature was identified as an independent predictor of patient survival in association with other clinicopathological factors in univariate [hazard ratio (HR): 3.0, 95% Confidence Interval (CI) 1.66–5.44, *p* = *2*.*7E-4*] and multivariate [HR: 2.88, 95% CI 1.57–5.2, *p* = 0.001] analyses. Subset analyses demonstrated that the signature could predict patients who would attain complete or partial remission or no-response to first-line chemotherapy. Pathway analyses revealed that the signature was regulated by HIF1α and TP53 and included nine HIF1α-regulated genes, which were highly expressed in non-responders and partial remission patients than in complete remission patients. We present the 97-gene signature as an accurate prognostic predictor of overall survival and chemoresponse. Our signature also provides information on potential candidate target genes for future treatment efforts in ovarian cancer.

## Introduction

Ovarian cancer has the highest mortality rate of all gynecological cancers and the fifth highest mortality rate of all cancers in the world^1^. Because the early stage of the disease is asymptomatic, ovarian cancer is rarely diagnosed until it reaches a late stage^2^. Primary debulking surgery followed by first-line chemotherapy (platinum-taxane) is the standard treatment in advanced ovarian cancer^3, 4^. Approximately 70% of patients achieve complete remission (CR) after the current standard treatment; however, 30% to 40% of these patients relapse within 12 months^5, 6^. The overall 5-year survival still remains at 30%^7, 8^, necessitating the need for better prognostic prediction of chemoresponse in ovarian cancer.

Clinicopathological characteristics such as histological grade and the International Federation of Gynecology and Obstetrics (FIGO) staging system are considered as the most important prognostic indicators in ovarian cancer. However, they are insufficient for predicting survival time and chemoresponse^9, 10^. Currently, there is no fully validated and clinically applicable test to guide treatment decisions in ovarian cancer.

Several gene signatures associated with overall survival and response to chemotherapy have been established in ovarian cancer^11–15^, but none are in clinical use yet due to discrepancies in technology and disease heterogeneity. Therefore, identification of a prognostic gene signature that captures the key pathways/regulators leading to platinum and taxane resistance in ovarian cancer is critical for prediction of patient outcome and overall survival (OS).

In this study, we established a novel prognostic gene signature to distinguish low- and high-risk patients, using a gene expression profiling technique, by analyzing seven microarray datasets and one RNA-seq dataset. Furthermore, we assessed the association of the identified prognostic gene signature with clinicopathological factors and investigated whether it could predict chemoresponse. Our findings suggest an optimal treatment based on the molecular characteristics of ovarian cancer patients.

## Results

Seven microarray datasets (GSE49997, GSE14764, GSE26712, GSE63885, GSE19829, GSE30161, The Cancer Genome Atlas – TCGA) and one TCGA RNA-seq dataset were used in this study. Data selection was based on availability of clinicopathological information such as OS time and event, grade, stage, chemoresponse, and related chip types. Detailed information for these datasets is described in the Materials and Methods (Supplementary Table S1).

**Figure 1:**
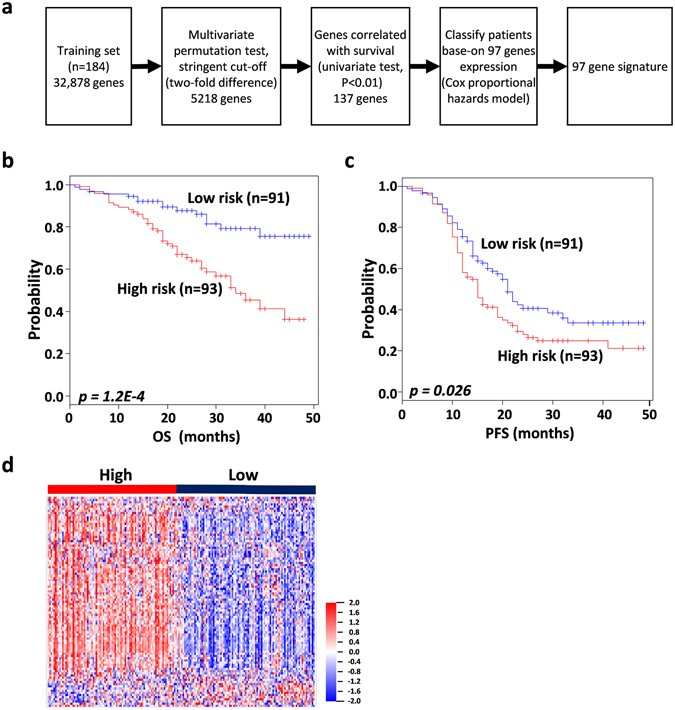
Training set survival analysis (GSE49997). (**a**) Schematic overview of the procedure used in the construction of the 97 gene signature based on gene expression data. (**b**) Kaplan-Meier plots depicting overall survival (OS) into low and high risk groups in the training data set predicted by compound covariate predictor (CCP). (**c**) Kaplan-Meier plots depicting progression- free survival (PFS) of patients into low and high risk groups in the training data set. The *p values* were computed by the log-rank test. (**d**) The heat map of the median centered 97 genes expression profiles (red, relative high expression; blue, relative low expression) between low and high risk groups.

The end point of this study was the identification of a gene signature prognostic of overall survival after first-line chemotherapy in both low- and high-risk groups. GSE49997 was used as the training dataset. A supervised method of risk prediction, with a stringent threshold cut off (*p* < 0.01 and 2-fold difference) was applied to the training dataset. After filtering for probe set intensity, 5,218 genes were analyzed using univariate Cox proportional hazard regression (*p* < 0.01) and 137 genes associated with OS were identified. However, only 97 genes were measured in the training dataset and all validation datasets (Fig. 1a). The 97-gene signature defined a threshold that split the training dataset into low- and high-risk groups with 91 and 93 patients, respectively. To evaluate the prognosis, Kaplan-Meier survival curves were plotted and the log-rank test showed a significant difference in OS (*p* = *1*.*2E-4;* Fig. 1b) and progression-free survival (PFS) (*p* = *0*.*0*2*6*; Fig. 1c). The expression pattern of the 97-gene signature divided low- and high-risk patients into two clusters (Fig. 1d).

### Prognostic significance of the 97-gene signature and clinical relevance in the training dataset

To investigate the prognostic significance of the 97-gene signature in association with other covariates in ovarian cancer, univariate and multivariate Cox regression analyses were performed on variables including age, stage, and grade in the training dataset. Both in univariate and multivariate analyses, the 97-gene signature showed the strongest prognostic ability in association with OS (HR 3.008, 95% Cl 1.6–5.4, *p* = *2*.*7E-4* and HR 2.885, 95% CI 1.57–5.27, *p* = *0.001*, respectively; Table 1).

**Table 1:** Univariate and multivariate Cox proportional hazard regression analyses in the training dataset.

Variable	Univariate analysis			Multivariate analysis		
	HR	95% CI	P value	HR	95% CI	P value
Age	1.687	0.826–3.444	0.020	1.729	1.009–2.963	0.046
Stage	1.762	0.990–3.138	0.054	1.186	0.626–2.248	0.600
Grade	0.698	0.488–2.999	0.049	1.449	1.008–2.082	0.045
97 gene signature	3.008	1.662–5.445	2.7E-4	2.885	1.579–5.271	0.001

Current clinicopathological factors in ovarian cancer including age, stage and grade analyzed in association with the 97 gene signature in the training dataset.

Kaplan-Meier plots were made to show which patients were low- or high-risk, based on grade and FIGO staging system. Grades 1 & 2 were combined as low grade (well differentiated, *p* = *0*.*0191;* Supplementary Fig. S1a) and grade 3 was considered high grade (poorly differentiated, *p* = *8*.*1E-5*; Supplementary Fig. S1b). Because ovarian cancer is usually discovered at a late stage, most of the patients in the training dataset were in stage III (*p* = *0.00185*; Supplementary Fig. S1c) and stage IV (*p* = *0.0191*; Supplementary Fig. S1d).

### Validation of the 97-gene signature in independent datasets

**Figure 2:**
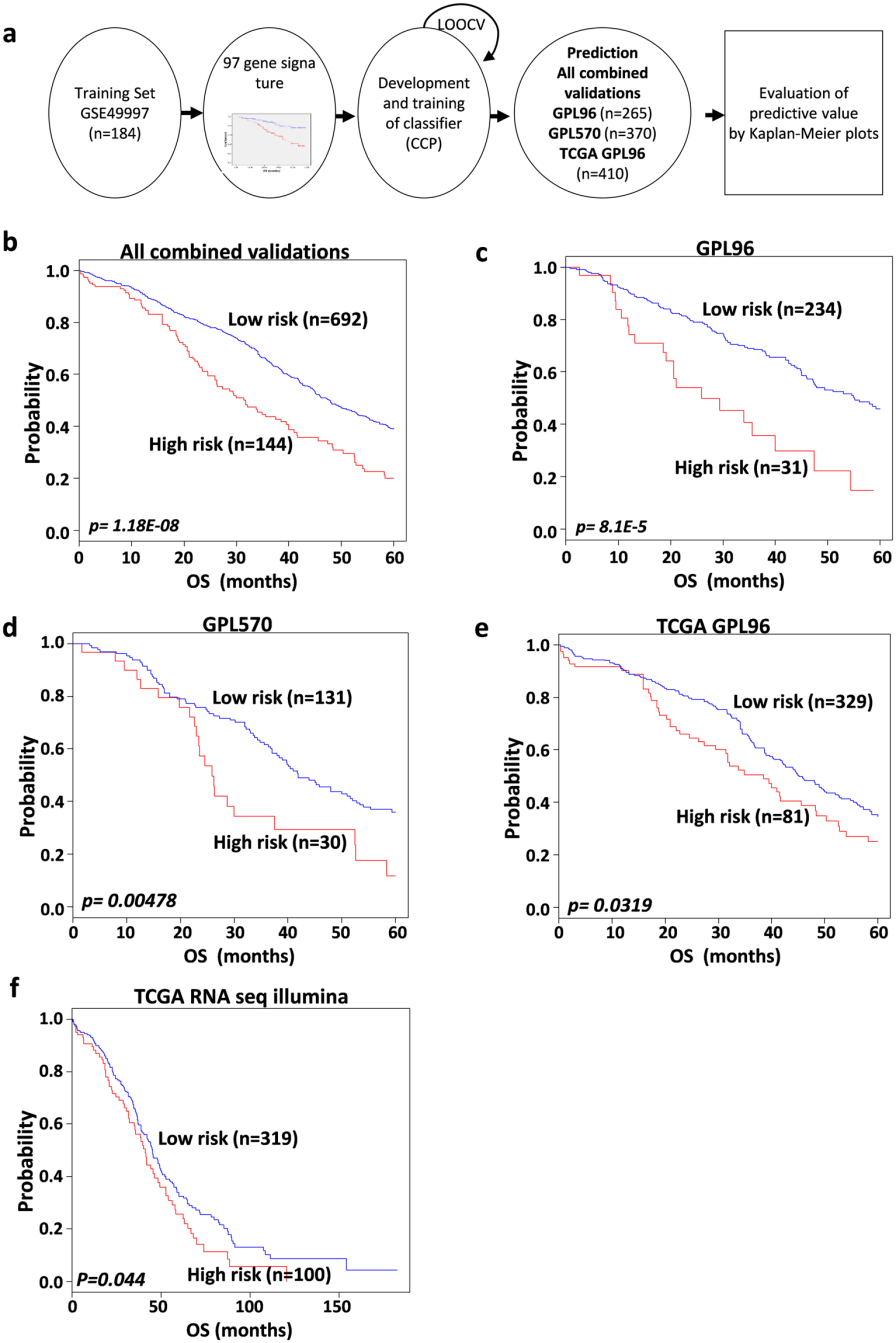
The prognostic significance of the 97 gene signature in all combined validation data sets. (**a**) A schematic overview of the strategy used in the construction of the prediction model and evaluation of predicted outcomes in the merged validation data sets. (**b**) Overall survival Kaplan-Meier plots stratified patients into low and high risk groups in all combined validation data set. (**c**) Overall survival Kaplan-Meier curves dichotomized patients into low and high risk groups in GPL96 data set (GSE14764, GSE26712). (**d**) Kaplan-Meier curves stratified patients in GPL 570 data set into low and high risk groups (GSE 63885, GSE19829, and GSE30161). (**e**) Overall survival Kaplan-Meier curves of patients in TCGA GPL96 data set classified into low and high risk groups. (**f**) Kaplan-Meier for overall survival of patients classified into low and high risk groups in TCGA RNA seq data set, predicted by compound covariate predictor (CPP). The *p values* were computed by log-rank test.

To further assess outcome prediction, we combined all of the validation datasets (total of 836 patients) to evaluate the robustness of the 97-gene signature. We further validated the prognostic significance of the gene signature in an external TCGA RNA-seq dataset. A flow chart of the procedure used to validate external datasets is depicted in Fig. 2a. Leave-one-out cross-validation (LOOCV) specificity and sensitivity used was 0.972 and 0.932 in Compound Covariate Prediction (CCP), respectively. The gene signature significantly stratified patients in all combined validations into low- and high-risk groups (*p* = *1*.*1*8*E-*0*8*; Fig. 2b). Most of the independent datasets had a small number of patients. Therefore, to assess the generalizability of our gene signature, validation datasets were merged according to chip type as follows: GSE14764 and GSE26712 as *GPL96*; GSE63885, GSE19829 and GSE30161 as GPL570; and TCGA GPL96. The merged validation datasets’ Kaplan- Meier plots predicted significant differences in prognosis: GPL96 (*p* = *8*.*1E-5*; Fig. 2c), GPL570 (*p* = *0.00478*; Fig. 2d), TCGA GPL96 (*p* = *0.0319*; Fig. 2e) and TCGA RNA-seq as an independent validation (*p* = *0.044*; Fig. 2f). Kaplan-Meier graphs for each independent validation dataset demonstrated a decrease in the overall survival of the high-risk group versus the low-risk group, but some datasets were statistically insignificant because of the small number of patients (Supplementary Fig. S3).

### Prognostic subclassification of patients in relation to current clinical covariates

To investigate the prognostic significance of the 97-gene signature in association with stage and grade in all validation datasets, univariate and multivariate Cox regression analyses were performed. In both univariate and multivariate analyses, the 97-gene signature proved to be a stronger predictor of OS than grade and stage (HR 1.778, 95% CI 1.4–2.2, *p* = *1.2 E-6*; and HR 1.749, 95% Cl 1.385–2.209, *p* = *2*.*6E-6* respectively; Table 2).

**Table 2:** Univariate and multivariate Cox proportional hazard regression analyses in all combined validation datasets.

Variable	Univariate analysis			Multivariate analysis		
	HR	95% CI	P value	HR	95% CI	P value
Stage	1.383	1.170–1.634	1.4E–4	1.314	1.290–2.184	0.001
Grade	1.798	1.379–2.344	1.4E-5	1.678	1.108–1.559	1.1E-4
97 gene signature	1.778	1.409–2.242	1.2E-6	1.749	1.385–2.209	2.6E-6

Current clinicopathological factors in ovarian cancer including Figo stage and grade analyzed in association with the 97 gene signature in all combined validation data set. (HR) hazard ratio; (Cl) confident interval; FIGO, International Federation of gynecology obstetrics; OS, overall survivor.

**Figure 3:**
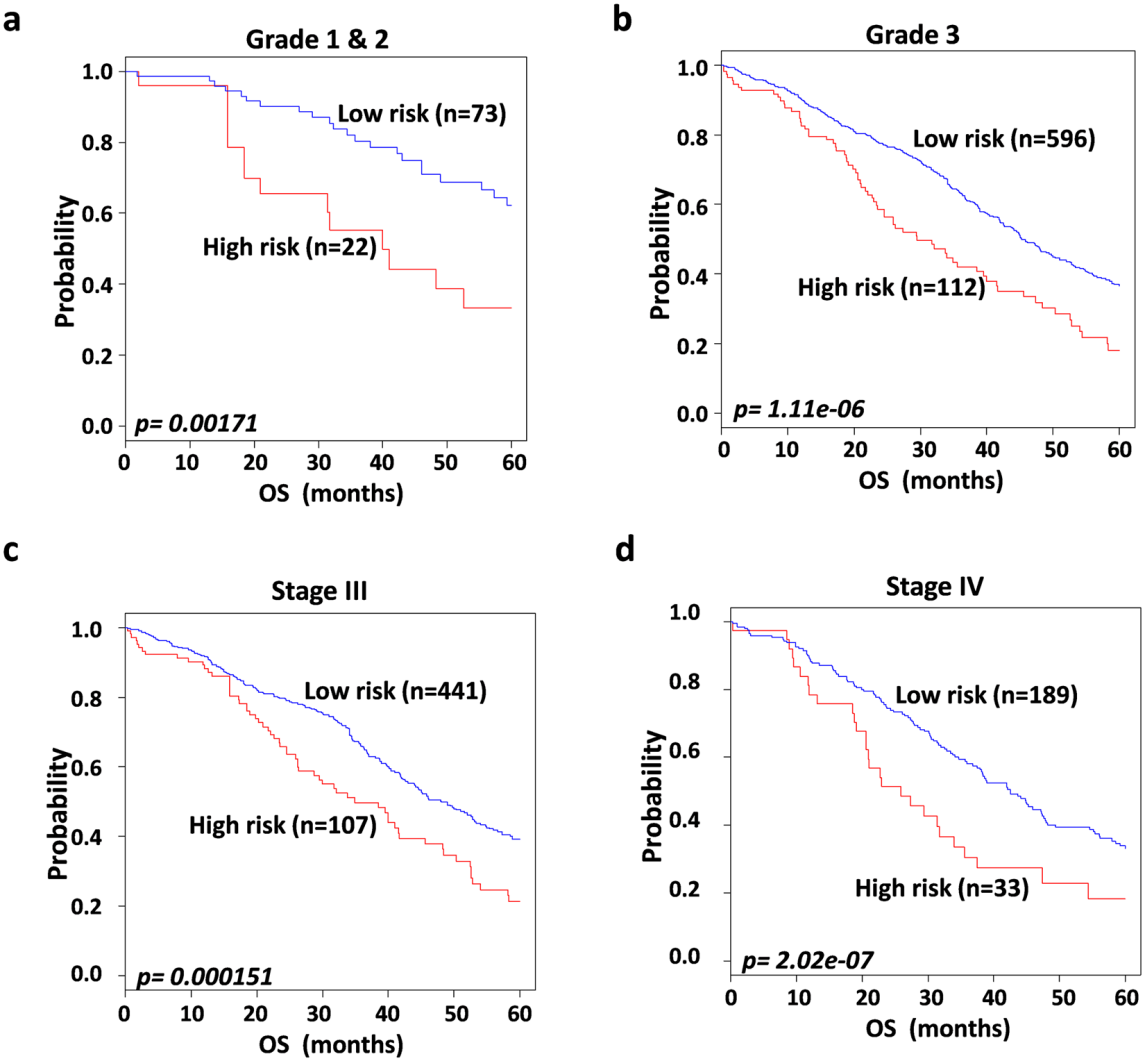
Prognostic significance of the 97 gene signature in relation to grade and FIGO stage in the combined validation data set. Patients from all combined validations merged according to grade and stage. Kaplan-Meier plots were predicted by compound covariate predictor (CPP). The *p values* were computed by the log-rank test. (**a**) Overall survival Kaplan-Meier plots of patients in low and high risk groups in grade 1 & 2. (**b**) Kaplan-Meier plots for overall survival of patients in low and high risk groups in grade 3. (**c**) Overall survival Kaplan-Meier plots showing patients in low and high risk groups in FIGO stage III. (**d**) Kaplan-Meier plots depicting overall survival of patients into low and high risk groups in FIGO stage IV. FIGO; International Federation of gynecology and obstetrics.

To assess whether the 97-gene signature could stratify patients in the low grade into low- and high-risk groups, patients in grades 1 and 2 were merged because only 5patients were in grade 1. The 97-gene signature significantly stratified patients into low- and high-risk groups (*p* = *0.00171*; Fig. 3a). The gene signature also separated patients in grade 3 into low- and high-risk groups (*p* = *1*.*11e-*0*6*; Fig. 3b).

Further, to evaluate whether the 97-gene signature could classify patients in each stage into low- and high-risk groups, all patients from the validation datasets were merged according to stage: I (n = 20), II (n = 27), III (n = 548) and IV (n = 222). The signature clearly stratified patients in stage III (*p* = *0*.*000151*; Fig. 3c) and stage IV (*p* = *2*.*02E-07;* Fig. 3d).

### Prognostic value of the 97-gene signature in association with chemoresponse

**Figure 4:**
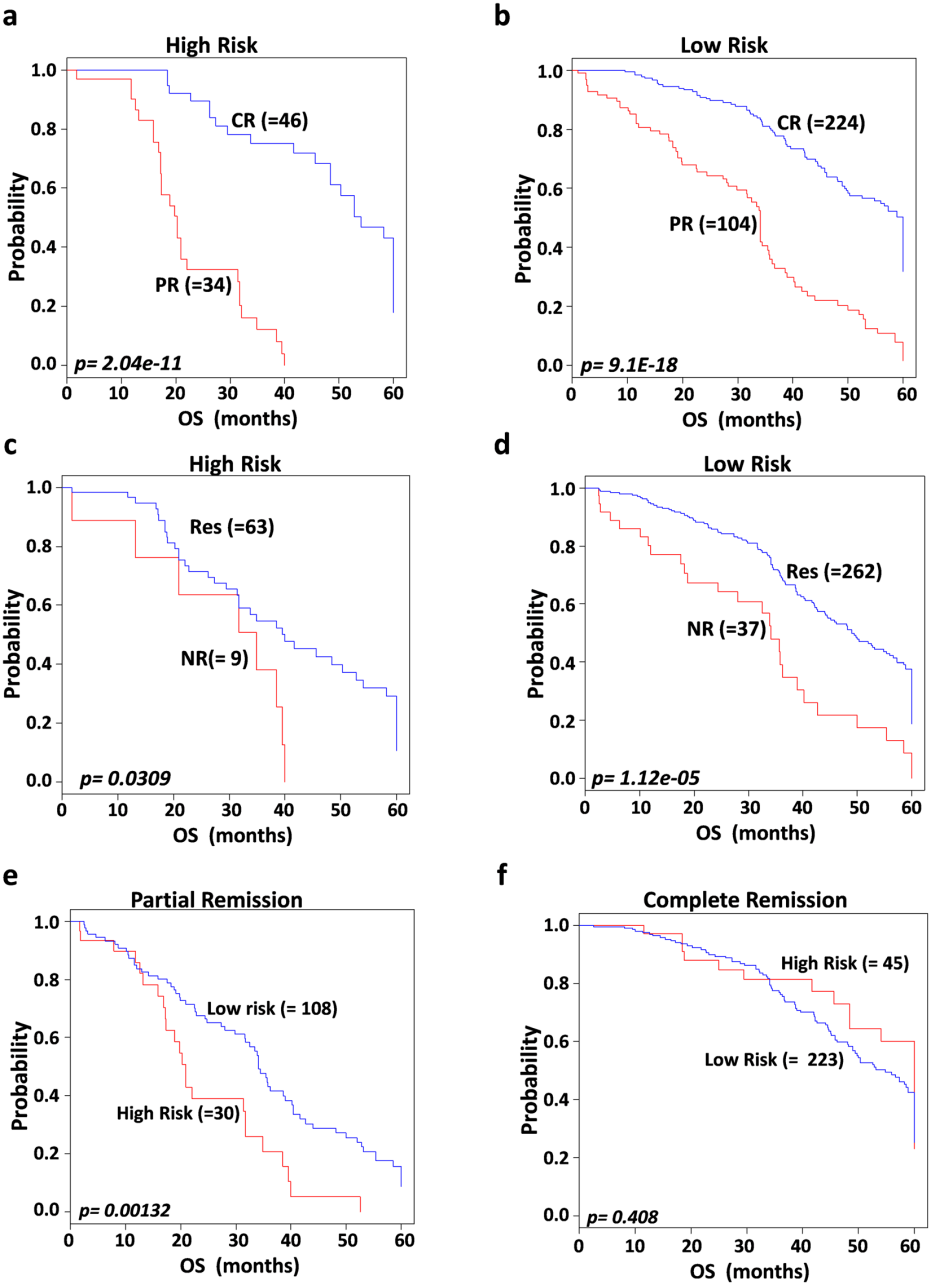
Significant association of the 97 gene signature with combined platinum and taxane based chemotherapy. Patients from the combined validation dataset with available first line chemotherapy information were included for the analysis predicted by compound covariate predictor (CPP). The *p values* were computed by the log rank test. (**a**) Overall survival Kaplan-Meier plots for patients in high risk group classified into complete and partial remission groups. (**b**) Kaplan-Meier overall survival analysis stratified patients in low risk group into complete or partial remission. (**c**) Overall survival Kaplan-Meier plots classified patients in high risk group into responders and non- responders. (**d**) Kaplan-Meir survival analyses stratified patients in low risk group into responders and non-responders to first line chemotherapy. (**e**,**f**) Kaplan-Meier plots depicting low and high risk in partial and complete remission subgroups, with patients in low risk group showing better prognosis. The *p values* were computed by log-rank test.

All patients in our study received first-line platinum and taxane combined chemotherapy. Patients were considered to be in complete remission (CR) if all detectable tumors disappeared after first-line chemotherapy, partial remission (PR) if the tumor significantly reduced in volume, and non-responder (NR) or stable disease (SD) if the tumor continued to grow or new tumors appeared. To evaluate whether our gene signature could accurately predict response to first-line chemotherapy, subset analyses were performed on TCGA (n = 326) and GSE30161 (n = 58) datasets, for which chemoresponse information was available. The signature clearly classified low-risk patients into CR and PR groups (*p* = *9*.*1E-18*, Fig. 4a) and high-risk patients into CR and PR groups (*p* = *2*.*04E-11*; Fig. 4b). The signature also dichotomized low- and high-risk patients into responders and non-responders (*p* = *1*.*12E-*0*5* and *p* = *0.0309*, respectively; Fig. 4c,d). To consolidate these results regarding chemosensitivity, we sorted both CR and PR case groups into low- and high-risk. Our gene signature showed that the low-risk group had better prognosis both in PR (Fig. 4e) and CR (Fig. 4f).

### Gene Ontology term enrichment analysis and visualization of the 97-gene signature

We performed Gene Ontology enrichment analysis in DAVID to identify biological functions of the genes in the 97-gene signature and identified 20 significant terms (biological processes), including metastasis, migration, growth factor binding, angiogenesis, regulation of apoptosis, and regulation of epithelial cell proliferation. False discovery rates were estimated using the procedure of Benjamin (*p* < 0.05, Supplementary Table S2).

### Transcription factors and pathways associated with the 97-gene signature

**Figure 5:**
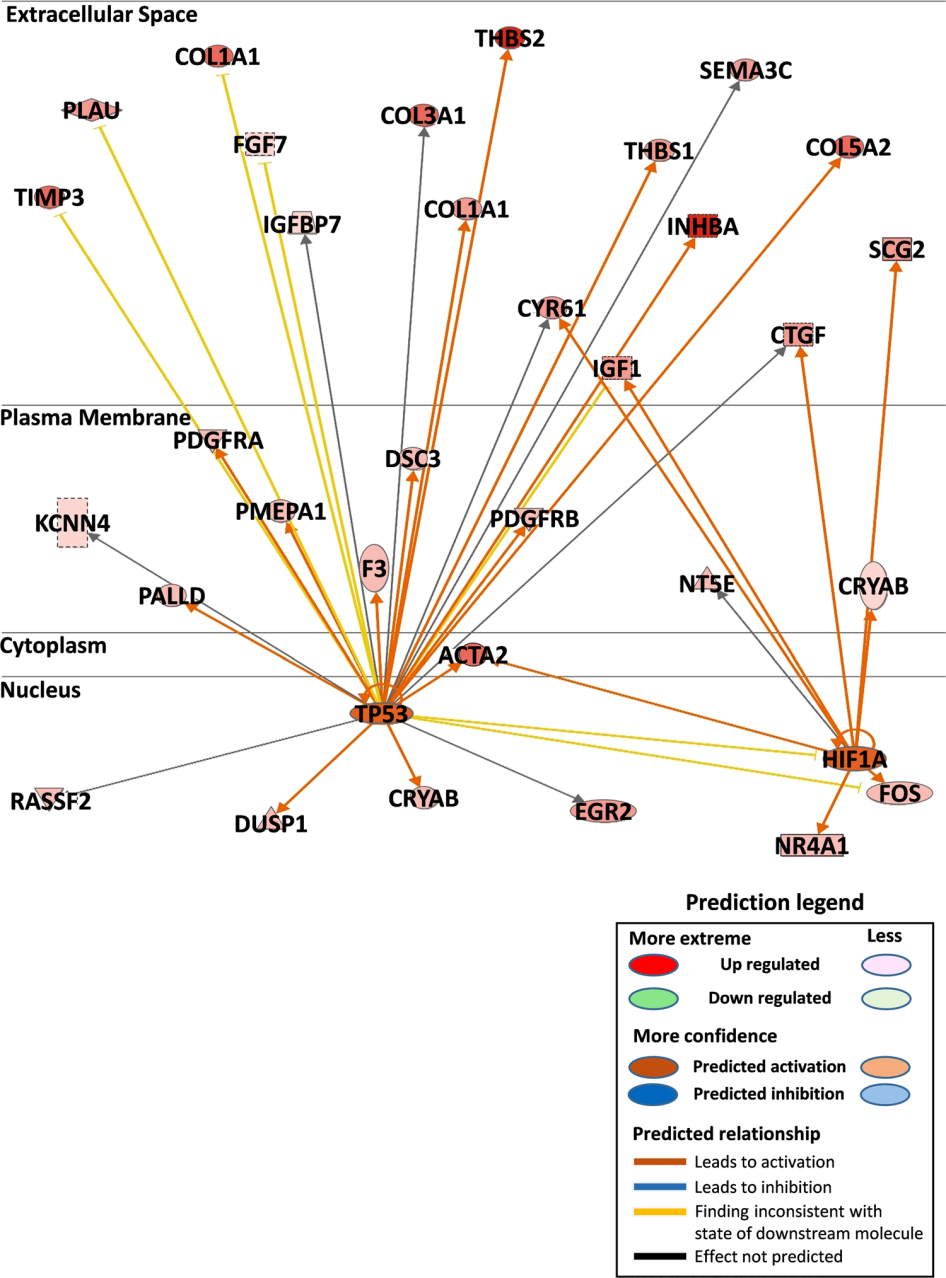
Significant association of the 97 gene signature with transcription factors TP53 and HIF1α. IPA analysis of gene networks from the 97 gene signature significantly associated with TP53 and HIF1α pathways. Identified gene networks were ranked to score (Z-score = 02). TP53, *p* = *0.000267* and HIF1α *p* = *0.00103*. IPA; Ingenuity Pathway Analysis. TP53; Tumor protein p53. HIF1α; Hypoxia-inducible factor 1-alpha.

We investigated transcription factors and pathways associated with our gene signature, using Ingenuity Pathway Analysis software. TP53 and HIF1α were found to be the most activated transcription factors. TP53 regulated genes involved in cell proliferation, angiogenesis, and metastasis (Fig. 5), and HIF1α regulated important genes involved in tumor cell growth, angiogenesis, and blood vessel morphology (Fig. 4). Furthermore, we showed that genes from the 97-gene signature that were involved in angiogenesis and metastasis were the most upregulated (Supplementary Fig. S3). We also demonstrated interactions using the STRING database to elaborate physical and functional association amongst the genes in the signature (Supplementary Fig. S4).

### Significant association of the HIF1α-regulated genes with platinum and taxane

**Figure 6:**
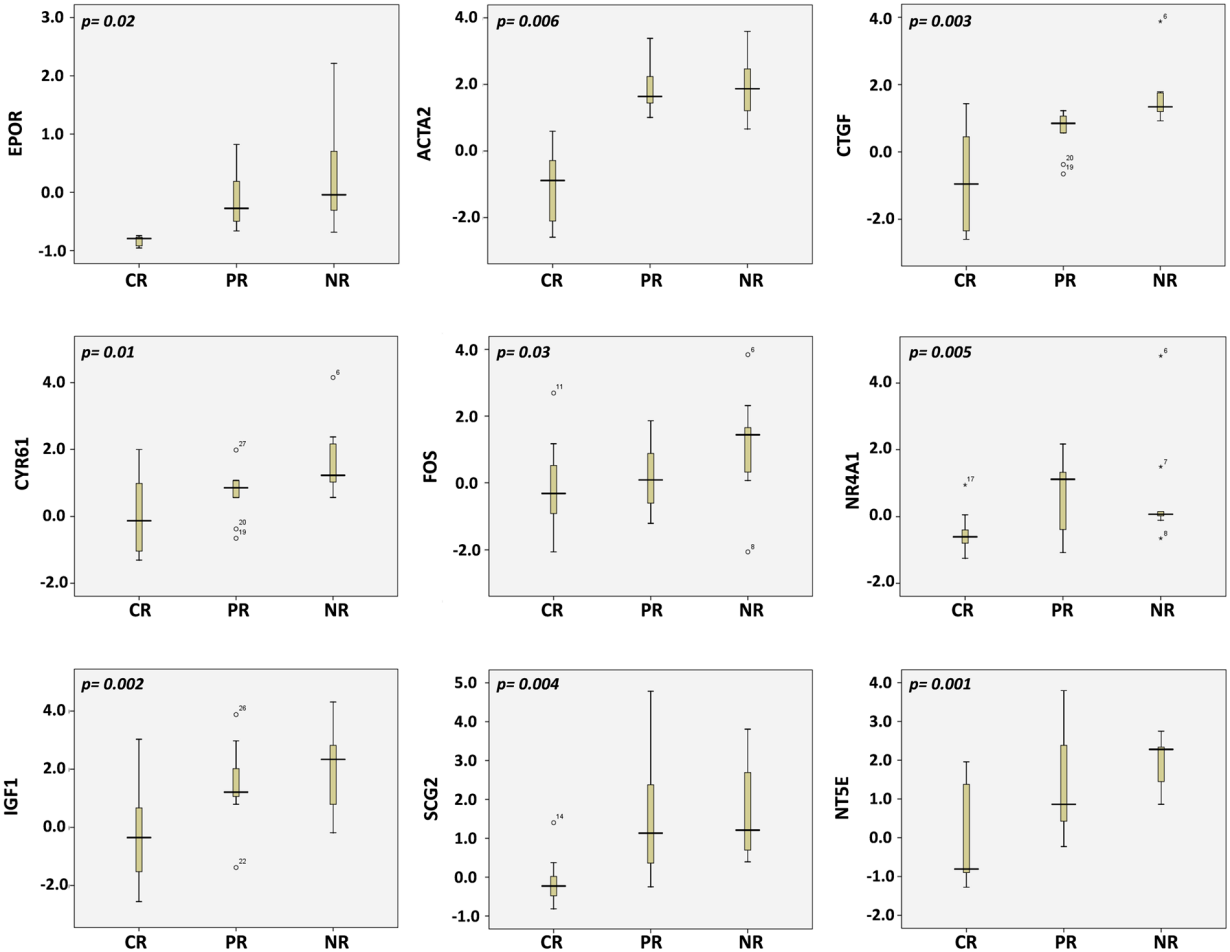
HIF1α nine genes box plots in CR, PR and NR. Individual box plots depicting expression levels for HIF1α regulated genes in complete remission, partial remission and non-responders groups. The left vertical represents the expression of each gene in SPSS and the *p values* are indicated on the graphs. CR; Complete Remission. PR; Partial Remission. NR; No-responder. SPSS; Statistical Package for the Social Sciences.

To understand the molecular mechanisms behind the three chemoresponse groups CR, PR, and NR, individual box plots for each of the nine genes were conducted (Fig. 6). HIF1α-regulated genes were found to be highly expressed in NR and PR but less expressed in CR cases.

## Discussion

**S**everal reports have attempted to show that gene signatures are capable of predicting individual clinical outcomes in ovarian cancer^16–23^. Prognostic gene signatures based on chemoresistance, molecular subtyping, and debulking tumors in ovarian cancer have been reported by analysis of homogenous datasets^24–27^. However, none of these are in clinical use yet because of difficulties caused by ovarian cancer heterogeneity. Hence, precise prognostic prediction for chemoresponse and mortality after first-line chemotherapy remains a critical issue in ovarian cancer treatment. Here, we developed a novel risk classification system based on a 97-gene signature from ovarian cancer patients^27^. Patients from various study groups were merged in order to get insight into distinctive endpoints of ovarian cancers and to overcome challenges reported by previous studies, such as homogeneity or small patient size. A supervised method was used to construct the gene signature that was then refined by LOOCV. Furthermore, a meta-analysis approach based on six Affymetrix microarray datasets (n = 836) and one RNA-seq dataset (n = 419) was applied to validate the prognostic significance of the gene signature in association with overall survival. The reproducibility of the gene signature was improved using only Affymetrix validation datasets. Univariate and multivariate analyses with adjustment for clinical parameters showed a significant association of our prognostic gene signature with survival rate. The 97-gene signature was also able to predict chemoresponse in both low- and high-risk patients. Moreover, our signature revealed nine HIF1α-regulated genes to be predictive of response to first-line platinum and taxane chemotherapy in ovarian cancer.

In clinical oncology, grade and stage are the current diagnostic criteria. However, the heterogeneity observed in therapeutic response within the same grade or stage indicates that histological classification is not an adequate predictor of survival^9^. In our study, univariate and multivariate Cox proportional hazards regression analyses of clinical variables based on OS in both the training and validation datasets also indicted that the 97-gene signature was a more reliable prognostic indicator than current clinical variables. Taken together, we think that the integration of the 97-gene signature with grade and stage could be a better approach for determining patient prognosis in clinical practice.

The precise prediction of chemoresponse would greatly improve ovarian cancer patient prognosis by guiding chemotherapy choices. However, it is difficult to determine the response to first-line chemotherapy because of the complexity of ovarian cancer genetic heterogeneity^20^. Several investigators previously reported gene signatures associated with chemoresponse in ovarian cancer^28, 29^. However, most of these studies were performed on small numbers of patients and were only prognostic, not predictive, of first-line chemotherapy response. The 97-gene signature could predict patients who would attain complete or partial remission after first-line chemotherapy in both low- and high-risk groups. In addition, the gene signature was also predictive of non-responders (NR) to first-line chemotherapy in both low- and high-risk groups. This predictability of our gene signature could benefit PR and NR patients by directing the choice of effective alternate and/or additional therapeutic options early in the disease.

Some of the genes in the 97-gene signature have been reported to play critical roles in biological processes, including angiogenesis, metastasis, and proliferation in ovarian cancer. These genes include: CLIP, COL3A1, COL10A1, LAMB1, INHBA, NBL, F3, PDGFRA, PDGFRB, PLAU, ROR2, SCG2, and ZFHX4^30^; COL11A1, COL5A2, THBS2, TIMP3 and SINAI2^31^; EGR2^32, 33^ and FOS^34^. Moreover, we investigated transcription factors included in the 97-gene signature. Consistent with previous studies, p53^35, 36^ and HIF1α^22^ were the upregulated transcription factors. However, a previous study reported that a large phase III trial of adenoviral wild-type p53 delivery combined with taxol and carboplatinum did not show any positive results in ovarian cancer^37^. To date, there is no accepted explanation of why this trial failed^38^. It is well known that the p53 signaling pathway has many links to ovarian cancer and is triggered by various stress signals^39^.

We investigated our novel nine HIF1α-regulated genes (ACTA2, EPOR, CTGF, FOS, SCG2, IGF1, CYR61, NT5E and NR4A). Interestingly, these genes had higher expression in NR and PR but lower expression in CR. Several studies reported ACTA2 upregulation in high-grade ovarian cancer^40, 41^. Western blot analysis by McBroom JW demonstrated increased EPOR expression in multiple ovarian cancer cell lines^42^. CTGF was reported to be a target therapeutic gene in high-grade ovarian cancer^43^. FOS has also been reported as a molecular predictor of progression and survival in ovarian cancer^44^. SCG2 was mentioned in a systematic review of genes reported to play a role in chemotherapy resistance in ovarian cancer^30^. IGF1 is well known to be involved in the development and progression of several types of tumors, including ovarian cancer^45^. Overexpression of CYR61 has been shown to be associated with development and poor prognosis of ovarian carcinomas^46^. NT5E-expressing cancers were reported to be resistant to platinum-based therapy^47^, while NR42A has been reported to be highly expressed in ovarian cancer tumors with poor prognosis^48^. We found that these HIF1α-regulated genes play significant roles in ovarian cancer first-line chemotherapy resistance, leading to disease progression and poor clinical outcome. These results suggest the possibility of developing and testing anti-HIF1α target based therapies for non-responders and partial remission patients.

In conclusion, we propose that this 97- prognostic gene signature could predict patient outcome in ovarian cancer. This gene signature can stratify subgroups of ovarian cancers into low- and high-risk groups in a reliable and reproducible manner across combined and independent patient cohorts. Our data suggest that this classifier may have considerable clinical relevance, especially in predicting first-line chemoresponse. This study will enable additional efforts to move ovarian cancer signatures closer to clinical relevance, and not only offer insight into mechanisms of chemoresistance but also provide information on potential target genes for future therapies.

## Materials and Methods

Our evaluation was performed in four major steps: selection of both eligible genomic datasets and eligible prognostic models, transparent reimplementation of risk prediction models identified by literature review, selection of the gene signature from the training dataset, and multi-study validation of the selected gene signature using meta-analytic methods.

### Eligibility Criteria and Implementation of the Prognostic Model

All datasets that had prognostic value in epithelial ovarian cancer were considered. Dataset GSE49997 from Austria was selected as the training dataset. Although it had 204 patients, only 184 patients were used. Twenty patients were excluded because they lacked OS information (Supplementary Table S1). Probe set identifiers were translated into gene symbols^27^.

### Eligibility criteria for datasets and samples

Data in the public domain that provided expression information about primary patient tumors, OS time and event, and related chip types were assessed. The analysis was repeated to exclude samples that did not fit the above mentioned criteria.

### Information sources

Prognostic models were identified through PubMed searches. The following terms were used to search PubMed: (“ovarian cancer” OR “Ovary tumor” OR “ovary carcinoma” OR “ovarian tumor” OR “ovarian carcinoma” OR “microarray” OR “expression array” OR gene expression AND ovarian cancer gene signature).

All datasets used were downloaded from National Center for Biotechnology Information Gene Expression Omnibus database (http://www.ncbi.nlm.nih.gov/geo) and Cancer Genome Atlas database (http://cancergenome.nih.gov/). The robust multi-array averaging (RMA) method was used to average raw files. Seven different datasets were used with a total of 1,020 patients (Supplementary Table S1).

Datasets GSE14764 (n = 80; GPL96, Charite University Hospital, Berlin)^49^, GSE26712 (n = 185; GPL96, National Cancer Institute, USA)^24, 50^, GSE63885 (n = 75; GPL570, Memorial Cancer Center and Institute of Oncology, Poland)^26^, GSE19829 (n = 28; GPL570, Harvard Medical School, USA)^25^, GSE30161 (n = 58; GPL570, University of Virginia)^51^, and TCGA (n = 410; GPL96) were merged (n = 836) for a combined validation (Supplementary Table S1). In addition, RNA-seq data from TCGA (n = 419; Illumina) was used as an external validation dataset. (https://genome-cancer.ucsc.edu).

### Development of the prognostic gene expression signature

To develop a prognostic gene expression signature, dataset GSE49997 was used as the training dataset. Overall survival and gene expression data were combined to build a gene expression profiling based survival classifier^52^. The 5,218 genes measured were filtered by at least 2 absolute values of log_2_ scale, which represented the same gene expression level. Univariate cox proportional hazard regression (*P* < 0.01) was then used to identify an OS associated gene expression signature from the discovery data set. For prognosis prediction, genes from the survival signature were applied in the survival risk prediction analysis. The prognostic index was computed by the formula Σ_*i*_ *w*_*i*_ *x*_*i*_ − 0.467581 where *w*_*i*_ and *x*_*i*_ are the weight and logged gene expression for the *i*-th gene. A sample was predicted as high (low) risk if their prognostic index was larger than (smaller than or equal to) the median prognostic index of −0.038061. Initially, 137 genes associated with survival were discovered in the training dataset, however, only 97 genes were measured by both U133 plus 2.0 and U133A, and so these genes were used in all validations as the 97 gene signature (Supplementary Table S3).

### Validation of the prognostic signature

Six independent datasets were used to validate the gene signature. Gene expression from different datasets was adjusted individually by subtracting the median expression value from all samples before merging. CCP was used as the class prediction algorithm to further refine this model and sub-stratify the predicted outcomes. Robustness was estimated by the misclassification rate that was determined in the training dataset during LOOCV^21^.

Kaplan-Meier survival analyses were performed after classification of patients into two risk groups, and Chi-square (χ^2^) and log-rank tests were used to evaluate the survival risk between the two predicted subgroups of patients. Univariate and multivariate Cox proportional hazard regression analyses were performed to evaluate independent prognostic factors associated with survival. The gene signature, age, tumor grade, and FIGO stage were employed as covariates.

### Pathway analysis

The Database for Annotation, Visualization, and Integrated Discovery (DAVID) bioinformatics tool was used for Gene Ontology (GO) biological process enrichment analysis, *P* values of less than 0.05 was used to define significant pathways. Ingenuity Pathways Analysis (IPA) was also used for transcription factor and gene network analyses (http://www.ingenuity.com)^53^. Identified gene networks were ranked to score (Z-score = 02).

### STRING analysis

To predict protein-protein interactions, Search Tool for Retrieval of Interacting Genes/Proteins (STRING) database V10.0 was used (http://www.string-db.org/). Proteins were linked based on the following six criteria: neighborhood, gene fusion, co-occurrence, co- expression, experimental evidence, and existing databases^54^.

### Statistical methods for microarray data

Microarray data and heatmaps were analyzed using BRB-Array Tools Version 3.0 (http://linus.nci.nih.gov/BRB-ArrayTools.html)^55^. All other statistical analyses were accomplished in the R language environment (www.r-project.org) and Statistical Package for Social Sciences (SPSS) software (version 20, SPSS Inc., Chicago, IL, USA). In all statistical analyses, *P* values of less than 0.05 was considered significant.

## Electronic supplementary material

Supplementary Information
