# Supplementary material for: The Prognostic 97 Chemoresponse Gene Signature in Ovarian Cancer
Source: Sci Rep. 2017 Aug 29;7:9689. doi: 10.1038/s41598-017-08766-5 (PMC5575202; doi:10.1038/s41598-017-08766-5)
Supplement: Supplementary file 1 — Supplementary Information [file 41598_2017_8766_MOESM1_ESM.pdf]

The Prognostic 97 Chemoresponse Gene Signature in Ovarian Cancer

Abel Matondo[1,*] Yong Hwa Jo[2,*], Muhammad Shahid[2, 5], Tae Gyu Choi[2], Minh Nam Nguyen[2] , Ngoc Ngo Yen Nguyen[1] , Salima Akter[2], Insug Kang[2], Joohun Ha[2], Chi Hoon Maeng[3], Si-Young Kim[3], Ju-seog Lee[4], Jayoung Kim[5], and Sung Soo Kim[2, †]

[1]Department of Biomedical Science, Graduate School, Kyung Hee University

[2]Department of Biochemistry and Molecular Biology, Medical Research Center for Bioreaction to Reactive Oxygen Species and Biomedical Science Institute, School of Medicine, Kyung Hee University, Seoul, Republic of Korea

[3]Department of Medical Oncology and Hematology, School of Medicine, Kyung Hee University, Seoul, Republic of Korea

[4]Department of Systems Biology, Division of Cancer Medicine, University of Texas MD Anderson Cancer Center, Houston, Texas, USA

[5]Departments of Surgery and Biomedical Sciences, Cedars-Sinai Medical Center, Los Angeles, CA, USA

[*] These authors contributed equally to this work

[†] To whom correspondence should be addressed at:

Sung Soo Kim, MD, PhD

Department of Biochemistry and Molecular Biology, School of Medicine, Kyung Hee University, 26 Kyungheedae-ro, Dongdaemun-gu, Seoul 02447, Republic of Korea.

Tel: +82-2-961-0524; Fax: 82-2-959-8168; E-mail: sgskim@khu.ac.kr.

Contents:

Supplementary Fig. S1. Grade and FIGO stage subset analyses of the training data set.

Supplementary Fig. S2. Prognostic significance of the 97 signature in independent validation data sets

Supplementary Fig. S3. Genes involved in angiogenesis and metastasis.

Supplementary Fig. S4. Gene network in the 97 gene signature.

Supplementary Table S1. Datasets and clinical information.

Supplementary Table S2. Go term of the 97 gene signature.

Supplementary Table S3. The 97 gene signature.

Supplementary Fig S1

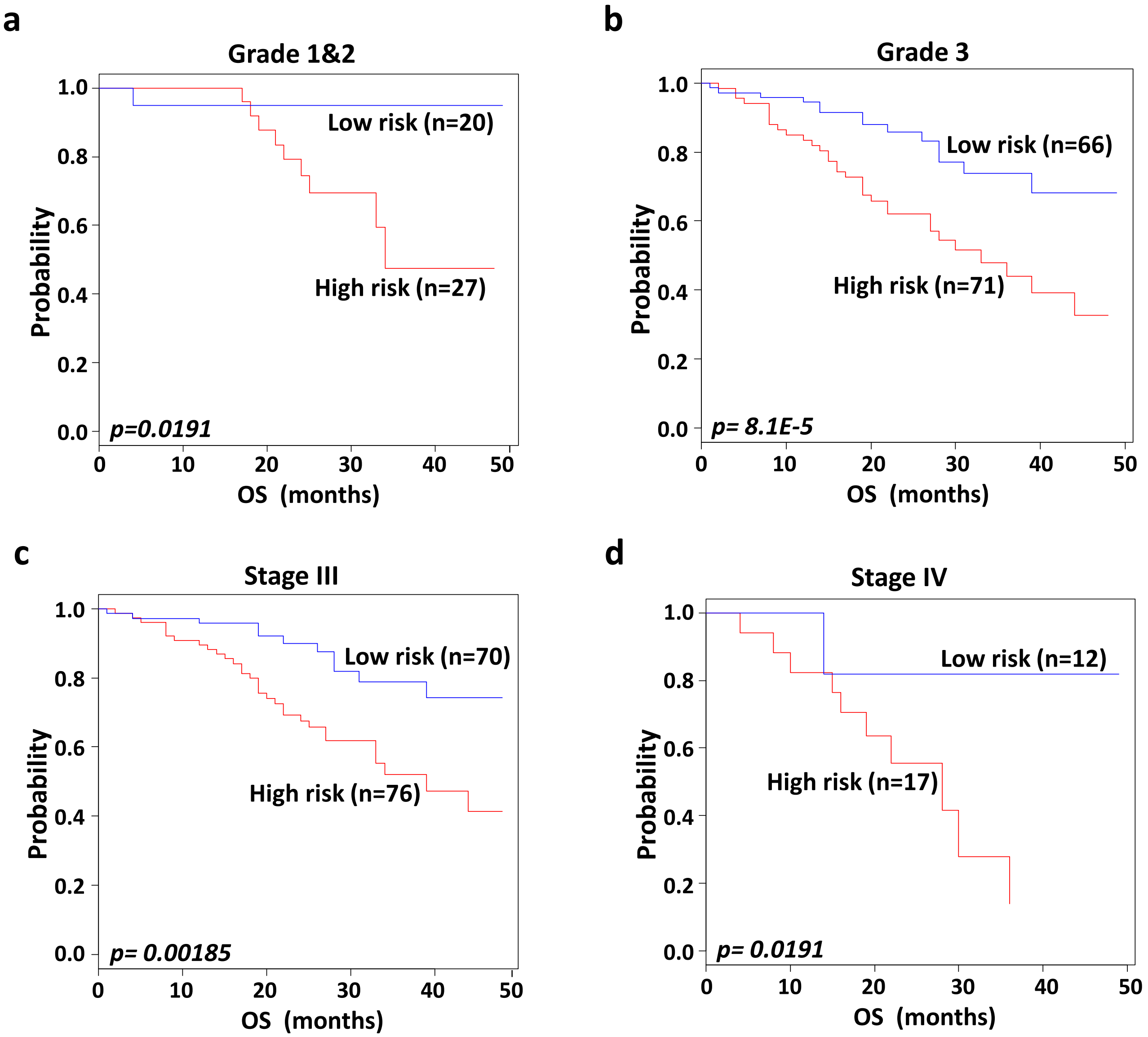

Supplementary Fig. S1. Grade and FIGO stage Subset analyses of the training data set. (a) Patients in grade 1&2 combined in the training set stratified into high and low risk groups. **(b)** Patients in grade3 in the training set stratified into low and high risk groups. **(c)** and **(d)** Patients in stages Ⅲ and Ⅳ in the training set classified into two groups.

Supplementary Fig S2.

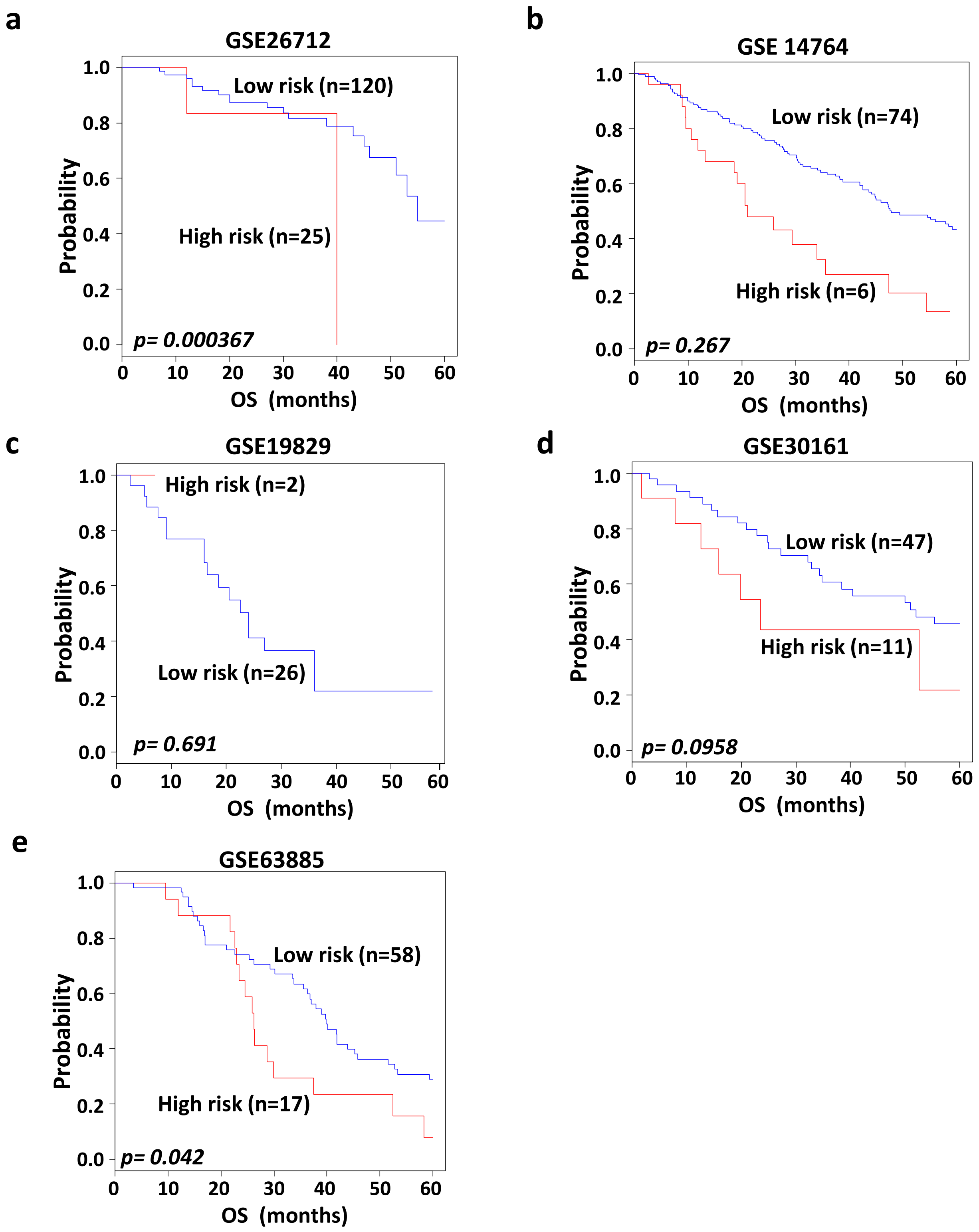

Supplementary Fig. S2. The prognostic significance of the 97 gene signature in combined validation data sets signature

(a) Kaplan-Meier curves if patients in GSE26712. **(b)** Kaplan-Meier curved of patients in GSE14764. **(c)** Kaplan-Meier curved of patients in GSE19829. **(d)** Kaplan-Meier curved of patients in GSE30161. **(e)** Kaplan-Meier curved of patients in GSE63885. Patients in high risk group showed poor prognosis. The *p values* were computed by log-rank test.

Supplementary Fig S3.

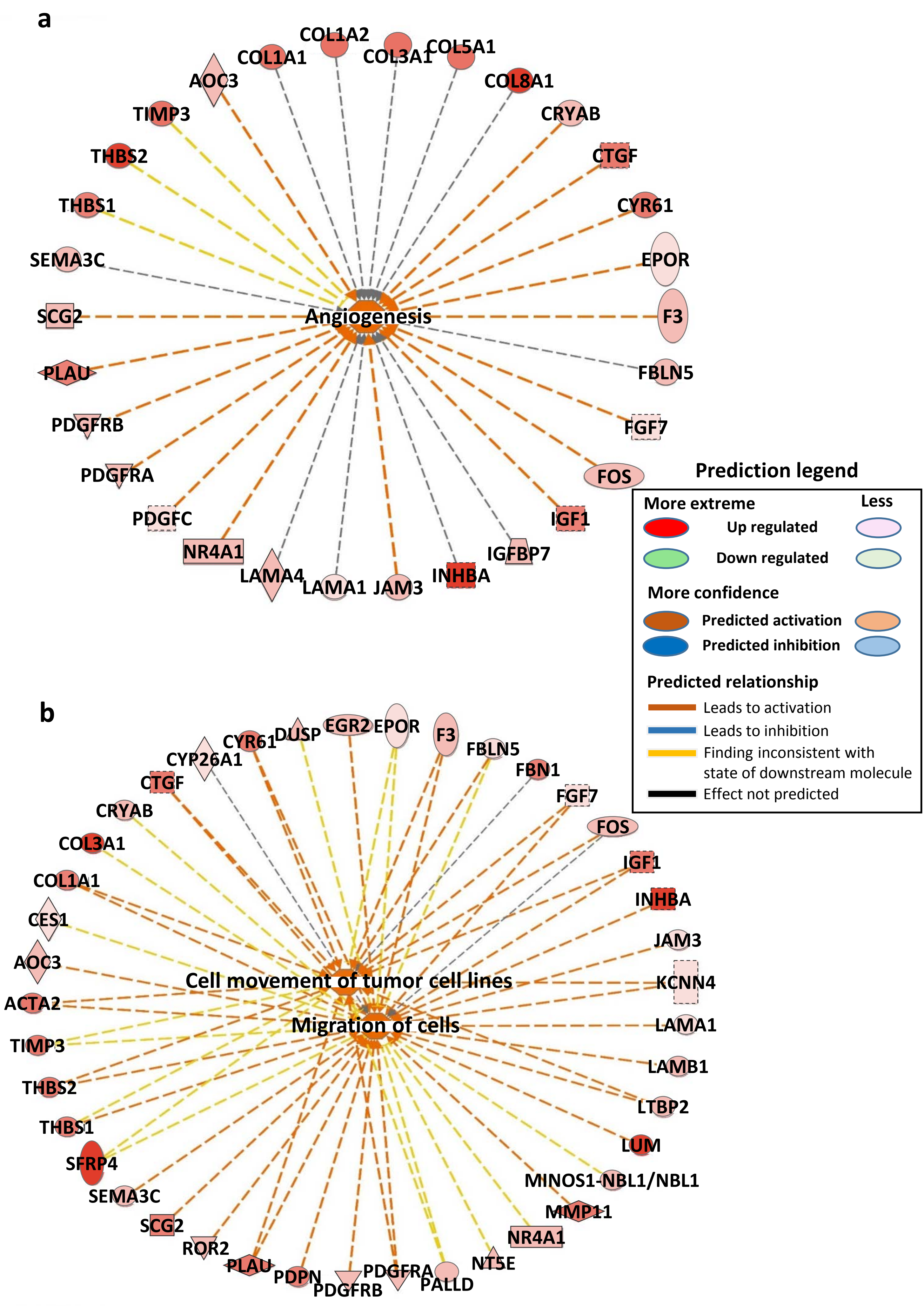

Supplementary Fig. S3. Genes involved in angiogenesis and metastasis. IPA upstream genes significantly associated with angiogenssis and metastasis pathways. Identified gene networks were ranked to score (Z-score=02). Angiogenesis *p=1.37E-01*, Metastasis *p=2.90E-01*.

Supplementary Fig S4.

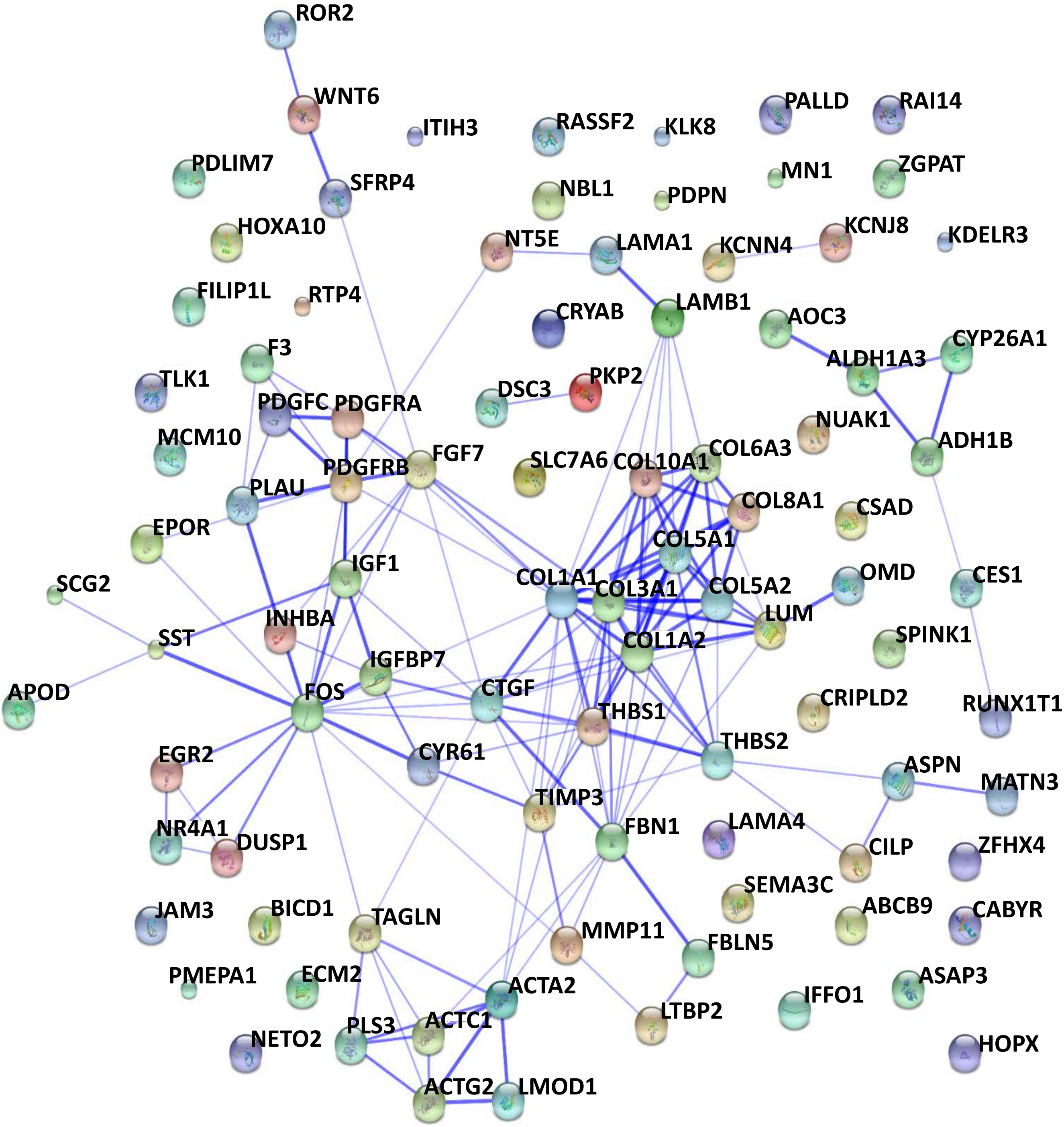

Supplementary Fig. S4. Gene network.
Interaction map was generated using String database. *p value* was indexed at 0.05.

Supplementary Table S1.

GEO Number	Origin/Year	Chip type	GPL	Reference
GSE49997	AUSTRIA 2013	Abi Human genome survey	GPL2986	Dietmar
GSE14764	GERMANY 2009	HG-U133A	GPL96	Jan
GSE26712	USA 2011	HG-U133A	GPL96	Micheal
GSE63885	POLAND 2011	HG-U133_Plus_2	GPL570	Kartarzyna
GSE19829	USA 2010	HG-U133_Plus_2	GPL570	Panagiotis
GSE30161	USA 2012	HG-U133_Plus_2	GPL570	Youngchul
TCGA	N/A	HG-U133A	GPL96	N/A

GSE, GEO data sets number prefixes. Abi Human genome survey, a chip type from applied Biosystems; HG-U33A and HG-U133_Plus_2, chip sets from Affymetrix.

Characteristics	Training dataset	Validation datasets					
	GSE49997	GSE14764	GSE26712	GSE63885	GSE19829	GSE30161	TCGA
**Number of patients (Patients used)**	204 (184)	80 (80)	195 (185)	101 (75)	80 (28)	66 (58)	422 (410)
**Median Age (years)**	57	-			65	62	58
**Median follow-up (months)**	41.485	35	38.28	36.8	35	33.65	30
**FIGO stage**							
**0**	0	0					0
Ⅰ	0	8		-			10
Ⅱ	9	1		2	2		20
Ⅲ	146	69	37	63	22	53	307
Ⅳ	29	2	148	10	4	5	63
**N/A**	0	0		-	-	-	10

**Grade**							
**1**	-	3					2
**2**	-	23		9			52
**1&2**	47	-			-	6	
**3**	137	54	185	48	28	52	347
**4**		-		18			1
**N/A**							8
**Chemotherapy**							
**Yes**	All	All	All	All	All	All	All
**CR**	-	-	-	-	-	33	231
**PR**	-	-	-	-	-	25	95
**NR**	-	-	-	-	-	-	40
**OS event**	56	21	102	55	17	36	223
**Recurrence**	118	-		49	13	48	236
**PFS: Median**	16		-	7.4	20.5	32	8.4

Clinical characteristics of patients analyzed. Training data set (GSE49997) and combined validation data sets.

Supplementary Table S2. Go term of the 97 gene signature.

Term	Function	Count	P value	Genes
GO:0044420	extracellular matrix part	15	1.19E-14	LUM, FBN1, COL3A1, CILP, COL5A2, TIMP3, COL5A1, LAMA1, LAMA4, COL6A3, COL1A2, COL1A1, COL8A1, LAMB1, COL10A1
GO:0005581	collagen	9	3.38E-11	LUM, COL6A3, COL3A1, COL1A2, COL1A1, COL8A1, COL5A2, COL5A1, COL10A1
GO:0030198	extracellular matrix organization	11	6.27E-10	CRISPLD2, FBLN5, LUM, COL3A1, COL1A2, PDGFRA, COL1A1, ECM2, COL5A2, COL5A1, CYR61
GO:0019838	growth factor binding	11	9.02E-10	LTBP2, CTGF, IGFBP7, COL3A1, COL1A2, PDGFRA, PDGFRB, COL1A1, THBS1, COL5A1, CYR61
GO:0001944	vasculature development	12	3.16E-07	LAMA4, PDPN, CTGF, COL3A1, COL1A2, SEMA3C, COL1A1, THBS1, COL5A1, PLAU, CYR61, SCG2
GO:0030334	regulation of cell migration	10	8.03E-02.15E-067	LAMA1, LAMA4, PDPN, F3, PDGFRA, PDGFRB, IGF1, LAMB1, THBS1, SST
GO:0001568	blood cessl development	11	1.60E-0	LAMA4, CTGF, COL3A1, COL1A2, SEMA3C, COL1A1, THBS1, COL5A1, PLAU, CYR61, SCG2
GO:0030335	pogitive regulation of cell migration	7	3.69E-04	PDPN, F3, PDGFRA, PDGFRB, IGF1, LAMB1, THBS1
GO:0009725	response to hormone stimulus	10	6.30E-04	FOS, EGR2, DUSP1, CRYAB, IGFBP7, PDGFRA, COL1A1, THBS1, SST, TIMP3
GO:0032963	collagen metabolic process	4	0.001406677	COL3A1, COL1A1, COL5A1, MMP11
GO:0016477	cell migration	8	0.001938236	LAMA1, CTGF, PDGFRB, SEMA3C, THBS1, COL5A1, PLAU, SCG2
GO:0050679	positive regulation of epithelial cell proliferation	4	0.00257129	LAMA1, FGF7, IGF1, LAMB1
GO:0051674	localization of cell	8	0.002670886	LAMA1, CTGF, PDGFRB, SEMA3C, THBS1, COL5A1, PLAU, SCG2
GO:0042127	regulation of cell proliferation	13	0.003549597	FGF7, IGFBP7, IGF1, LAMA1, F3, PDGFRA, PDGFRB, PDGFC, THBS1, LAMB1, SST, PLAU, SCG2
GO:0008284	positive regulation of cell proliferation	9	0.003549597	LAMA1, FGF7, F3, PDGFRA, PDGFRB, IGF1, PDGFC, LAMB1, SCG2
GO:0048514	blood vessel morphogenesis	6	0.009098216	CTGF, SEMA3C, THBS1, PLAU, CYR61, SCG2
GO:0000302	response to reactive oxigene species	4	0.010596219	FOS, DUSP1, CRYAB, COL1A1
GO:0001525	angiogenesis	5	0.012448515	CTGF, THBS1, PLAU, CYR61, SCG2
GO:0040008	regulation of growth	7	0.017298975	INHBA, CTGF, CRYAB, IGFBP7, HOPX, IGF1, CYR61
GO:0042981	regulation of apoptosis	11	0.023090145	INHBA, DUSP1, CRYAB, ALDH1A3, F3, NR4A1, IGF1, THBS1, SST, TIMP3, SCG2

Top ranked 20 Enriched categories amongst the transcripts significantly expressed. The *p value* was determined by Fishers' extract test with Benjamin.

Supplementary Table S3. **The 97 gene signature.**

Row	Name	Accession	Symbol
1	ATP-binding cassette, sub-family B (MDR/TAP), member 9	NM_001243013	ABCB9
2	actin, alpha 2, smooth muscle, aorta	NM_001141945	ACTA2
3	actin, alpha, cardiac muscle 1	NM_005159	ACTC1
4	actin, gamma 2, smooth muscle, enteric	NM_001199893	ACTG2
5	alcohol dehydrogenase 1C (class I), gamma polypeptide	NM_000669	ADH1C
6	aldehyde dehydrogenase 1 family, member A3	NM_000693	ALDH1A3
7	amine oxidase, copper containing 3	NM_001277731	AOC3
8	apolipoprotein D	NM_001647	APOD
9	ArfGAP with SH3 domain, ankyrin repeat and PH domain 3	NM_001143778	ASAP3
10	asporin	NM_001193335	ASPN
11	bicaudal D homolog 1 (Drosophila)	NM_001003398	BICD1
12	calcium binding tyrosine-(Y)-phosphorylation regulated	NM_012189	CABYR
13	carboxylesterase 1	NM_001025194	CES1
14	cartilage intermediate layer protein, nucleotide pyrophosphohydrolase	NM_003613	CILP
15	collagen, type X, alpha 1	NM_000493	COL10A1
16	collagen, type I, alpha 1	NM_000088	COL1A1
17	collagen, type I, alpha 2	NM_000089	COL1A2
18	collagen, type III, alpha 1	NM_000090	COL3A1
19	collagen, type V, alpha 1	NM_000093	COL5A1
20	collagen, type V, alpha 2	NM_000393	COL5A2
21	collagen, type VI, alpha 3	NM_004369	COL6A3
22	collagen, type VIII, alpha 1	NM_001850	COL8A1
23	cysteine-rich secretory protein LCCL domain containing 2	NM_031476	CRISPLD2
24	crystallin, alpha B	NM_001289807	CRYAB
25	cysteine sulfinic acid decarboxylase	NM_001244705	CSAD
26	connective tissue growth factor	NM_001901	CTGF
27	cytochrome P450, family 26, subfamily A, polypeptide 1	NM_000783	CYP26A1
28	cysteine-rich, angiogenic inducer, 61	NM_001554	CYR61
29	desmocollin 3	NM_001941	DSC3
30	dual specificity phosphatase 1	NM_004417	DUSP1
31	extracellular matrix protein 2, female organ and adipocyte specific	NM_001197295	ECM2
32	early growth response 2	NM_000399	EGR2
33	erythropoietin receptor	NM_000121	EPOR
34	coagulation factor III (thromboplastin, tissue factor)	NM_001178096	F3
35	fibulin 5	NM_006329	FBLN5
36	fibrillin 1	NM_000138	FBN1

37	fibroblast growth factor 7	NM_002009	FGF7
38	filamin A interacting protein 1-like	NM_001042459	FILIP1L
39	FBJ murine osteosarcoma viral oncogene homolog	NM_005252	FOS
40	G protein-coupled receptor 124	NM_032777	GPR124
41	HOP homeobox	NM_001145459	HOPX
42	homeobox A10	NM_018951	HOXA10
43	intermediate filament family orphan 1	NM_001039670	IFFO1
44	insulin-like growth factor 1 (somatomedin C)	NM_000618	IGF1
45	insulin-like growth factor binding protein 7	NM_001253835	IGFBP7
46	inhibin, beta A	NM_002192	INHBA
47	inter-alpha-trypsin inhibitor heavy chain 3	NM_002217	ITIH3
48	junctional adhesion molecule 3	NM_001205329	JAM3
49	potassium inwardly-rectifying channel, subfamily J, member 8	NM_004982	KCNJ8
50	potassium intermediate/small conductance calcium-activated channel, subfamily N, member 4	NM_002250	KCNN4
51	KDEL (Lys-Asp-Glu-Leu) endoplasmic reticulum protein retention receptor 3	NM_006855	KDELR3
52	kallikrein-related peptidase 8	NM_001281431	KLK8
53	laminin, alpha 1	NM_005559	LAMA1
54	laminin, alpha 4	NM_001105206	LAMA4
55	laminin, beta 1	NM_002291	LAMB1
56	leiomodin 1 (smooth muscle)	NM_012134	LMOD1
57	latent transforming growth factor beta binding protein 2	NM_000428	LTBP2
58	lumican	NM_002345	LUM
59	matrilin 3	NM_002381	MATN3
60	minichromosome maintenance complex component 10	NM_018518	MCM10
61	matrix metallopeptidase 11 (stromelysin 3)	NM_005940	MMP11
62	meningioma (disrupted in balanced translocation) 1	NM_002430	MN1
63	neuroblastoma 1, DAN family BMP antagonist	NM_001204084	NBL1
64	neuropilin (NRP) and tolloid (TLL)-like 2	NM_001201477	NETO2
65	nuclear receptor subfamily 4, group A, member 1	NM_001202233	NR4A1
66	5 -nucleotidase, ecto (CD73)	NM_001204813	NT5E
67	NUAK family, SNF1-like kinase, 1	NM_014840	NUAK1
68	osteomodulin	NM_005014	OMD
69	palladin, cytoskeletal associated protein	NM_001166108	PALLD
70	platelet derived growth factor C	NM_016205	PDGFC
71	platelet-derived growth factor receptor, alpha polypeptide	NM_006206	PDGFRA
72	platelet-derived growth factor receptor, beta polypeptide	NM_002609	PDGFRB
73	PDZ and LIM domain 7 (enigma)	NM_005451	PDLIM7
74	podoplanin	NM_001006624	PDPN
75	plakophilin 2	NM_001005242	PKP2
76	plasminogen activator, urokinase	NM_001145031	PLAU

77	plastin 3	NM_001136025	PLS3
78	prostate transmembrane protein, androgen induced 1	NM_001255976	PMEPA1
79	retinoic acid induced 14	NM_001145520	RAI14
80	Ras association (RalGDS/AF-6) domain family member 2	NM_014737	RASSF2
81	receptor tyrosine kinase-like orphan receptor 2	NM_004560	ROR2
82	receptor (chemosensory) transporter protein 4	NM_022147	RTP4
83	runt-related transcription factor 1; translocated to, 1 (cyclin D-related)	NM_001198625	RUNX1T1
84	secretogranin II	NM_003469	SCG2
85	sema domain, immunoglobulin domain (Ig), short basic domain, secreted, (semaphorin) 3C	NM_006379	SEMA3C
86	secreted frizzled-related protein 4	NM_003014	SFRP4
87	solute carrier family 7 (amino acid transporter light chain, y+L system), member 6	NM_001076785	SLC7A6
88	serine peptidase inhibitor, Kazal type 1	NM_003122	SPINK1
89	somatostatin	NM_001048	SST
90	transgelin	NM_001001522	TAGLN
91	thrombospondin 1	NM_003246	THBS1
92	thrombospondin 2	NM_003247	THBS2
93	TIMP metallopeptidase inhibitor 3	NM_000362	TIMP3
94	tousled-like kinase 1	NM_001136554	TLK1
95	wingless-type MMTV integration site family, member 6	NM_006522	WNT6
96	zinc finger homeobox 4	NM_024721	ZFHX4
97	zinc finger, CCCH-type with G patch domain	NM_001083113	ZGPAT

The list of genes in the 97 gene signature, including full names, gene symbols and *p values*.
